# OsNRAMP3 Is a Vascular Bundles-Specific Manganese Transporter That Is Responsible for Manganese Distribution in Rice

**DOI:** 10.1371/journal.pone.0083990

**Published:** 2013-12-31

**Authors:** Meng Yang, Wan Zhang, Huaxia Dong, Yuanyuan Zhang, Kai Lv, Dujun Wang, Xingming Lian

**Affiliations:** National Key Laboratory of Crop Genetic Improvement and National Center of Plant Gene Research (Wuhan), Huazhong Agricultural University, Wuhan, China; University of South Florida College of Medicine, United States of America

## Abstract

Manganese (Mn) is an essential trace element for plants. Recently, the genes responsible for uptake of Mn in plants were identified in *Arabidopsis* and rice. However, the mechanism of Mn distribution in plants has not been clarified. In the present study we identified a natural resistance-associated macrophage protein (NRAMP) family gene in rice, *OsNRAMP3*, involved in Mn distribution. *OsNRAMP3* encodes a plasma membrane-localized protein and was specifically expressed in vascular bundles, especially in phloem cells. Yeast complementation assay showed that OsNRAMP3 is a functional Mn-influx transporter. When *OsNRAMP3* was absent, rice plants showed high sensitivity to Mn deficiency. Serious necrosis appeared on young leaves and root tips of the *OsNRAMP3* knockout line cultivated under low Mn conditions, and high Mn supplies could rescue this phenotype. However, the necrotic young leaves of the knockout line possessed similar levels of Mn to the wild type, suggesting that the necrotic appearance was caused by disturbed distribution of Mn but not a general Mn shortage. Additionally, compared with wild type, leaf Mn content in *osnramp3* plants was mostly in older leaves. We conclude that OsNRAMP3 is a vascular bundle-localized Mn-influx transporter involved in Mn distribution and contributes to remobilization of Mn from old to young leaves.

## Introduction

Manganese (Mn) is an essential metal nutrient in most organisms. In plants, Mn plays an important role in photosystem II and is a required cofactor for a variety of enzymes [1]. Mn deficiency can reduce plant growth and increase susceptibility to low temperature and pathogen infection [2]. Despite its importance, the amount of Mn required by a plant is relatively low; however, the capacity for Mn uptake always exceeds this requirement and excess Mn can be particularly toxic to plant growth [3]. In general, the uptake and detoxification of Mn is well balanced in plants. Many gene families have been identified as involved in Mn uptake or detoxification of excess Mn.

Much of our understanding on Mn uptake in plants comes from the complementation tests on yeast mutant strain *Δsmf1*, which is deficient in Mn uptake [4]. *SMF1*, a gene from natural resistance-associated macrophage protein (NRAMP) family in yeast (*Saccharomyces cerevisiae*), is responsive to the high-affinity Mn^2+^ accumulation into yeast cell. In plants, NRAMP family genes also contribute to Mn^2+^ transportation or translocation. *AtNRAMP1* and *OsNRAMP5* encode plasma-located proteins that are major high-affinity Mn transporters in *Arabidopsis* and rice (*Oryza sativa* L.), respectively [5], [6]. AtNRAMP3 and AtNRAMP4, two other NRAMP members in *Arabidopsis*, are targeted to vacuolar membrane and operate in the retrieval of Mn^2+^ from vacuoles in leaf mesophyll cells under Mn deficiency [7]. Shown to be functional orthologs of AtNRAMP3 and AtNRAMP4, TcNRAMP3 and TcNRAMP4 from the metal hyperaccumulator *Thlaspi caerulescens* are both tonoplast proteins and can complement the *Δsmf1* yeast mutant phenotype [8]. When expressed in yeast strains, *LeNRAMP1* and *LeNRAMP3* from tomato (*Lycopersicon esculentum*) are targeted to vesicle and tonoplast, respectively, and can retrieve the phenotype of yeast mutant *Δsmf1* [9].

Mn always shares the same transporters with iron (Fe) in plants [3], [5], [10]. ZIP [zinc-regulated transporter/iron-regulated transporter (ZRT/IRT)-related proteins] genes and YSL (yellow stripe 1-like) genes are two well-known transporter families of Fe in plants. In recent years, many genes from these two families have been shown capable of transporting Mn. For example, AtIRT1 is an *Arabidopsis* ZIP transporter with a broad substrate range, including Mn^2+^ [11]. Besides *AtIRT1*, six other ZIP genes (*AtZIP1*, *AtZIP2*, *AtZIP3*, *AtZIP5*, *AtZIP6* and *AtZIP9*) from *Arabidopsis* were recently shown to be functional in transporting Mn^2+^ in yeast [12]. Several members of the ZIP family from plant species other than *Arabidopsis* also showed the ability to transport Mn^2+^, including *LeIRT1* and *LeIRT2* from tomato, *MtZIP4* and *MtZIP7* from *Medicago truncatula*, and *HvIRT1* from barley (*Hordeum vulgare*) [13]–[15]. In rice, no ZIP genes have been shown to transport Mn^2+^, but two members from the YSL family (*OsYSL2* and *OsYSL6*) showed activity in Mn^2+^ transport [16], [17].

Unlike NRAMP and ZIP gene families, cation exchanger (CAX) genes in plants are always involved in tolerance of Mn toxicity. In *Arabidopsis*, six CAX genes have been identified. *AtCAX1* and *AtCAX2* were the first CAX genes reported in plants and are known calcium (Ca) transporters [18]. However, expression studies in tobacco (*Nicotiana tabacum*) and yeast showed that *AtCAX2* is also involved in Mn^2+^ transport [19]. The protein encoded by *AtCAX2* is targeted to vacuolar membrane and confers Mn^2+^ tolerance in *Arabidopsis*. Furthermore, in a yeast Mn-tolerance screen with an *Arabidopsis* cDNA library, *AtCAX2* was the only gene identified out of the 10^5^ transformants that could suppress the Mn toxicity phenotype [20]. However, other CAX genes in *Arabidopsis* did not show Mn^2+^ transport activity. But interestingly, when expressed in yeast with an N-terminally truncated form, *AtCAX5* could mediate Mn^2+^ transport, suggesting that CAX genes in *Arabidopsis* may control the Mn^2+^ transport activity by an autoregulatory region at the N-terminus [21]. Five CAX genes have been identified in rice. The OsCAX3 and N-terminal truncated OsCAX1a from rice could confer tolerance to Mn when expressed in yeast [22]. In addition to the CAX gene family, the endomembrane-type Ca-ATPase (ECA) gene family is another one involved in both Ca^2+^ and Mn^2+^ transport. In *Arabidopsis*, *AtECA1* and *AtECA3* showed Mn transport activity when expressed in yeast [23], [24]. Interestingly, protein encoded by *AtECA1* is mainly targeted on endoplasmic reticulum membrane and increases tolerance of plants to Mn toxicity; however, *AtECA3* encodes a Golgi-localized protein and is required in *Arabidopsis* under Mn-deficient conditions [25].

The cation diffusion facilitator (CDF) gene family also contributes to Mn tolerance. Four CDF genes (*ShMTP1*–*ShMTP4*), the first CDF genes identified in plants, were screened from *Stylosanthes hamate* for enhancing Mn^2+^ tolerance by expressing a cDNA library in yeast [26]. Further studies on *ShMTP1* suggested that it encoded a tonoplast protein in *Arabidopsis* and conferred Mn^2+^ tolerance through internal sequestration. There are 12 members in the CDF family in *Arabidopsis*, and proteins encoded by four of these genes (*AtMTP8*–*AtMTP11*) are closely related to the protein encoded by *ShMTP1* [27]. Furthermore, knockout of *AtMTP11* in *Arabidopsis* resulted in hypersensitivity to high Mn^2+^, suggesting that *AtMTP11* can confer plant Mn tolerance [27], [28].

Most knowledge concerning Mn in plants was obtained from studies focused on uptake and tolerance, but little is known about the mechanisms of distribution and translocation of Mn in plants. In this study, we identified another NRAMP gene from rice, *OsNRAMP3*, involved in Mn distribution. *OsNRAMP3* encoded a plasma membrane-localized protein with activity in transporting Mn and was expressed specifically in vascular bundles, especially phloem cells. Knocking out of *OsNRAMP3* in rice resulted in high sensitivity to Mn deficiency and disturbed Mn distribution in leaves. These data suggested that *OsNRAMP3* played an important role in Mn distribution in rice.

## Materials and Methods

### Plant Materials

The knockout line of *OsNRAMP3* and wild type were based on *O. sativa* L. ssp. *japonica* cv. DongJin background. The *osnramp3* mutant is a T-DNA insert mutant ordered from the POSTECH RISD database (http://www.postech.ac.kr/life/pfg/risd/) [29]. Promoter analysis and expression profile analyzed by real-time PCR were based on *O. sativa* L. ssp. *japonica* cv. Zhonghua 11.

### Subcellular Localization of OsNRAMP3

The coding sequence of *OsNRAMP3* for subcellular localization was amplified using cv. Zhonghua11 total cDNA as template and primers 5′-gactgaattcatgagcggcccaatgcaa-3′ (forward) and 5′-gactggtaccatcgagatcagaagcagttcgct-3′ (reverse), which were designed based on the sequence of LOC_Os06g46310.1 from the RGAP database (http://rice.plantbiology.msu.edu/). The PCR product was cleaved using EcoR1 and Kpn1 and ligated into pM999–GFP with correct direction. The construct was transformed into *Arabidopsis* mesophyll protoplasts and observed using a confocal laser scanning microscope (TCS SP2; Leica) after incubation at 22°C for 12–24 h [30].

### GUS Staining Assay

To construct the *OsNRAMP3*-promoter:GUS plasmid, 2.1 kb of genomic sequence located upstream of the *OsNRAMP3* initiation codon was amplified by PCR from cv. Zhonghua 11 genomic DNA using primers Pnr5-F (5′-agctgcagatgcgccaaaatactgaat-3′) and Pnr5-R (5′-tcggatcctgcaagaaccctcaagact-3′). The amplified promoter fragment was digested by BamH1 and Pst1 and introduced into vector pDX2181 in the correct direction [31]. The construct was transformed into cv. Zhonghua 11 by *Agrobacterium tumefaciens*-mediated transformation [32]. The transgenic plant tissues were incubated in X-Gluc strain buffer at 37°C for 4 h [33].

### Hydroponic Experiments

Hydroponic experiments were performed according to the standard rice culture solution (1.44 mM NH_4_NO_3_, 0.3 mM NaH_2_PO_4_, 0.5 mM K_2_SO_4_, 1.0 mM CaCl_2_, 1.6 mM MgSO_4_, 0.17 mM Na_2_SiO_3_, 50 µM Fe-EDTA, 0.06 µM (NH_4_)_6_Mo_7_O_24_, 15 µM H_3_BO_3_, 8 µM MnCl_2_, 0.12 µM CuSO_4_, 0.12 µM ZnSO_4_, 29 µM FeCl_3_ and 40.5 µM citric acid at pH 5.5) [34]. The solution was renewed every 5 d.

### RNA Extraction and Real-time PCR

Total RNA was extracted using TRizol reagent (Invitrogen). Of total RNA, 3 µg was used to synthesize the first-strand cDNAs in 20 µl of reaction mixture using M-MLV reverse transcriptase (Invitrogen) according to the manufacturer’s instructions. Real-time PCR was performed using the SYBR Premix Ex Taq™ (TaKaRa) with the following gene-specific primers for *OsNRAMP3*: rqNR3-F (5′-tcagcagcgaactgcttctgatct-3′) and rqNR3-R (5′-atcagctggctaactctttgggct-3′). The rice *Ubiquitin 5* gene was used as the internal control with the following primers: qUbq-F (5′-aaccagctgaggcccaaga-3′) and qUbq-R (5′-acgattgatttaaccagtccatga-3′). The real-time PCR reaction was performed on an Applied Biosystems 7500 PCR instrument.

### Elemental Analysis

Shoots and roots were harvested separately and roots were washed with distilled water twice before sampling. After drying at 80°C for 3 d, all samples were digested in 65% nitric acid in a MARS6 microwave (CEM) at a temperature gradient of 120–180°C for 45 min, and then diluted in deionized water. The metal contents of the samples were determined by inductively coupled plasma–mass spectrometry (ICP-MS; Agilent 7700 series, USA).

### Yeast Complementation Assay

The cDNA fragments containing an entire open reading frame of *OsNRAMP3* or *AtNRAMP1* were amplified by the following primers: ycNR3-F (5′-gactggtaccatgagcggcccaatgcaac-3′) and ycNR3-R (5′-ctgagaattcctaatcgagatcagaagcagttcg-3′) for *OsNRAMP3*; and ycAtNR1-F (5′-gactggtaccatggcggctacaggatctggac-3′) and ycAtNR1-R (5′-gactggtacctcagtcaacatcggaggtagat-3′) for *AtNRAMP1*. The fragments were firstly cloned into pEGM-T Easy vector (Promega) and digested with Kpn1 and EcoR1 for *OsNRAMP3* or EcoR1 only for *AtNRAMP1*, and then correctly introduced into vector *pYES2*. The resulting plasmids were transformed into yeast strains. The yeast strains used in this study were *Δsmf1* (Mat a; his3Δ1; leu2Δ0; met15Δ0; ura3Δ0; YOL122c::kanMX4) and its wild-type BY4741 (MATa; his3Δ 1; leu2Δ 0; met15Δ 0; ura3Δ 0). Yeast cells were transformed according to standard procedures (Invitrogen). *Δsmf1* complementation testing by drop-spotting assays was performed on synthetic defined (SD)-Ura medium, which contained 2% galactose, 0.67% yeast nitrogen base without MnSO_4_ (Krackeler Scientific Inc), 0.2% appropriate amino acid, 2% agar, 50 mM 2-morpholinoethanesulfonic acid and supplemented without or with 2.5, 5 or 10 µM ethylene glycol bis(2-aminoethyl) tetraacetic acid (EGTA) at pH 6.0.

For metal determination, yeast cells transformed with different vectors were grown for 30 h starting from OD600 of 0.01 on SD-Ura liquid medium supplemented with 0.02 µM Mn at pH 6.0. Galactose instead of glucose was used here for induction of the GAL promoter. Cells were harvested by centrifugation and washed three times with deionized water (MilliQ; Millipore). All samples were dried at 80°C for 3 d, and then used to determine metal contents by ICP-MS.

## Results

### Expression Profiles of *OsNRAMP3* in Different Tissues of Rice

**Figure 1:**
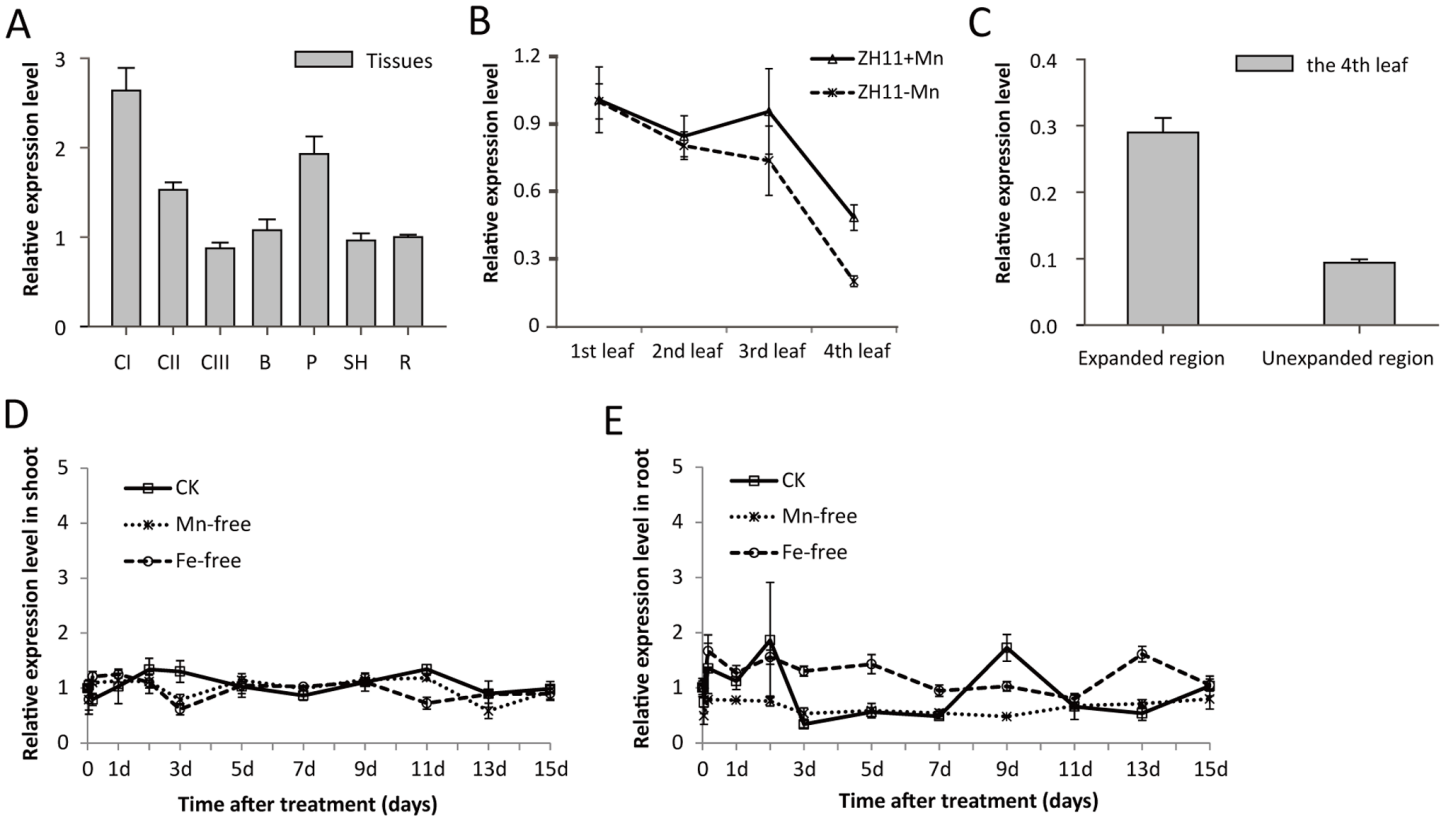
Expression analysis of *OsNRAMP3* by real-time RT-PCR. (A) The relative expression level of *OsNRAMP3* in different rice tissues. C1: first culm; CII: second culm; CIII: third culm; B: leaf blade; P: panicle; SH: leaf sheath; R: root. (B) Transcripts of *OsNRAMP3* of different leaves under different Mn conditions. The leaves detected here were sampled from the same tiller of rice plants – with the first leaf the oldest and the fourth leaf the youngest and partly wrapped in the leaf sheath. The plants were cultivated hydroponically under normal conditions for two weeks and then shifted to Mn-replete or Mn-free conditions for an additional one week. ZH11: cv. Zhonghua 11. (C) Expression analysis of *OsNRAMP3* in different regions of the fourth leaf. (D and E) Kinetics of the response of *OsNRAMP3* to Mn or Fe deficiency in shoot (D) and root (E) of rice plants. The plants were cultivated hydroponically under normal conditions for two weeks and then shifted to different conditions for treatment. Data are means ± SD (n = 3).

Seven different tissues from *O. sativa* L. ssp. *japonica* cv. Zhonghua 11 were used to investigate the expression pattern of *OsNRAMP3*. We found *OsNRAMP3* transcript levels to be similar in most tissues and slightly higher in panicles and the first culm (Figure 1A). We next detected transcript levels of *OsNRAMP3* in different leaves (Figure 1B). Four leaves from the same tiller were analyzed here, in which the first leaf was the oldest and the fourth leaf was youngest and partly wrapped in sheath. Interestingly, the expression level of *OsNRAMP3* increased with leaf age under both Mn-sufficient and Mn-deficient conditions. In addition, the fourth leaf showed a differential expression pattern of clearly lower *OsNRAMP3* expression in unexpanded compared to expanded regions (Figure 1C).

The NRAMP family genes in *Arabidopsis* are always induced by Fe or Mn deficiency [5], [35]. Time-course experiments under Mn and Fe deficiency were used to test the response of *OsNRAMP3* to Mn and Fe deficiency, respectively – real-time PCR revealed that *OsNRAMP3* was little affected by Mn or Fe deficiency (Figure 1D and 1E). In addition, expression did not respond to different Mn supplies (Figure S1).

### Identifying the *OsNRAMP3* Knockout Line

**Figure 2:**
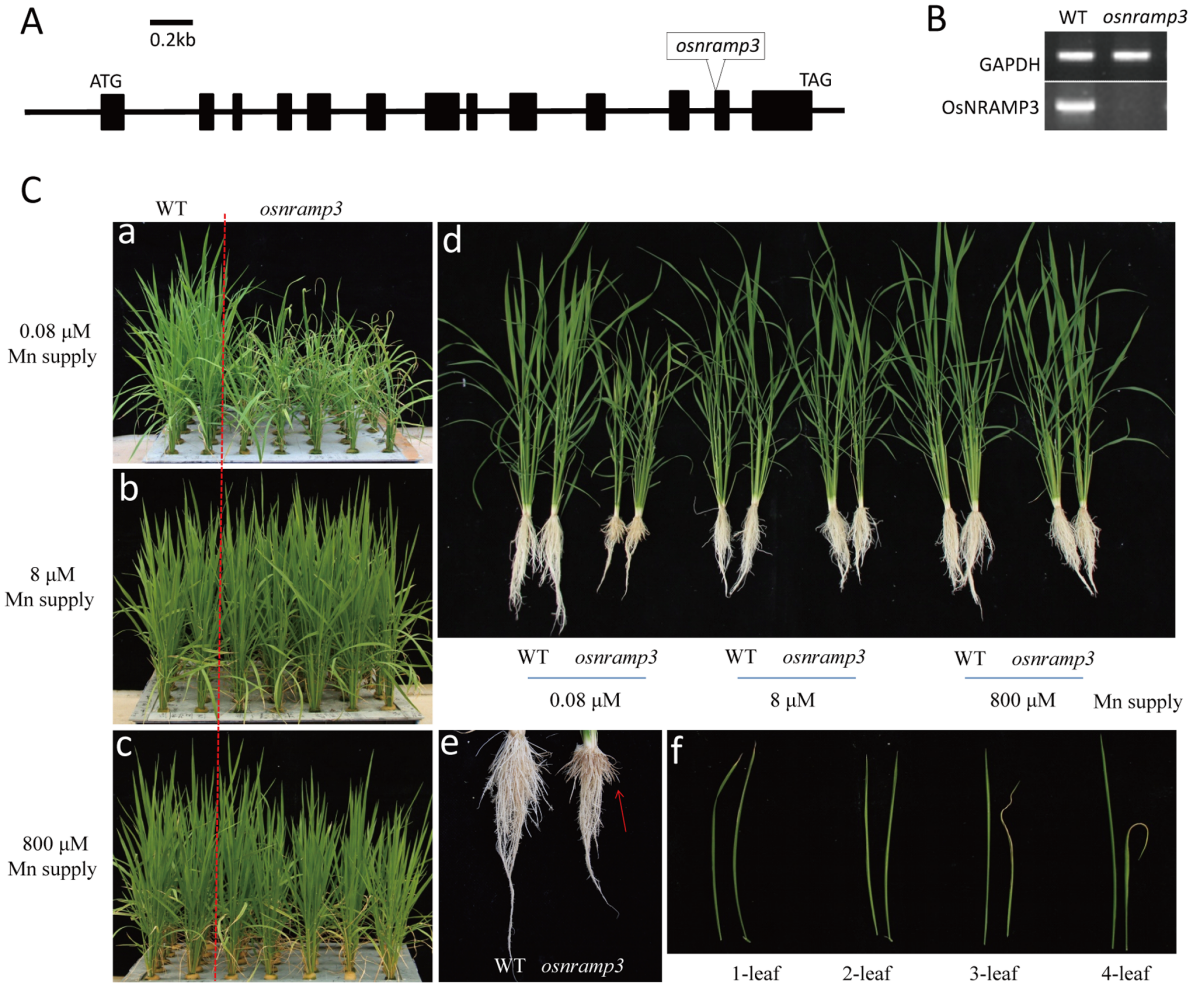
Identification of the knockout line of *OsNRAMP3*. (A) The structure of *OsNRAMP3*. *OsNRAMP3* contains 13 exons and 12 introns, and a T-DNA inserted into the 12^th^ exon of osnramp3 mutant plants. (B) The determination of accumulation of *OsNRAMP3* in *osnramp3* plants by RT-PCR. (C) Phenotypic analysis of knockout line of *OsNRAMP3*. The plants were cultivated hydroponically under normal conditions for two weeks and then shifted to different Mn supplies for an additional two weeks. Three levels of Mn were applied: 0.08 µM (a), 8 µM (b) and 800 µM (c). The general condition for growth of plants in the three Mn treatments was photographed (d). Careful observations were performed on roots (e) and leaves (f) of wild type and *osnramp3* plants at 0.08 µM Mn supply. The red arrow indicates the necrotic area that appeared in *osnramp3* roots. In (f), the left leaves are from wild type and the right leaves from *osnramp3*; 1–4 leaves were from the same tillers, with 1-leaf the oldest and 4-leaf the youngest.

*OsNRAMP3* contains 13 exons and 12 introns (Figure 2A), encoding a protein with 550 amino acids. To investigate the biological function of *OsNRAMP3*, we obtained a T-DNA insertion line from POSTECH [29]. The T-DNA was inserted into the twelfth exon. Transcripts of *OsNRAMP3* could not be detected in the line (Figure 2B), suggesting it was a knockout mutant of *OsNRAMP3*.

As NRAMP genes always play important roles in transporting Mn, we firstly investigated the response of *osnramp3* plants (the knockout line of *OsNRAMP3*) to different Mn supplies (Figure 2C, panels a–c). Interestingly, when plants were cultivated at low Mn supply (0.08 µM), *osnramp3* plants showed obviously reduced growth compared with wild type. Careful observation revealed serious necrosis in young leaves and roots tips of *osnramp3* plants, but not in older leaves (Figure 2C, panels d–f).

**Figure 3:**
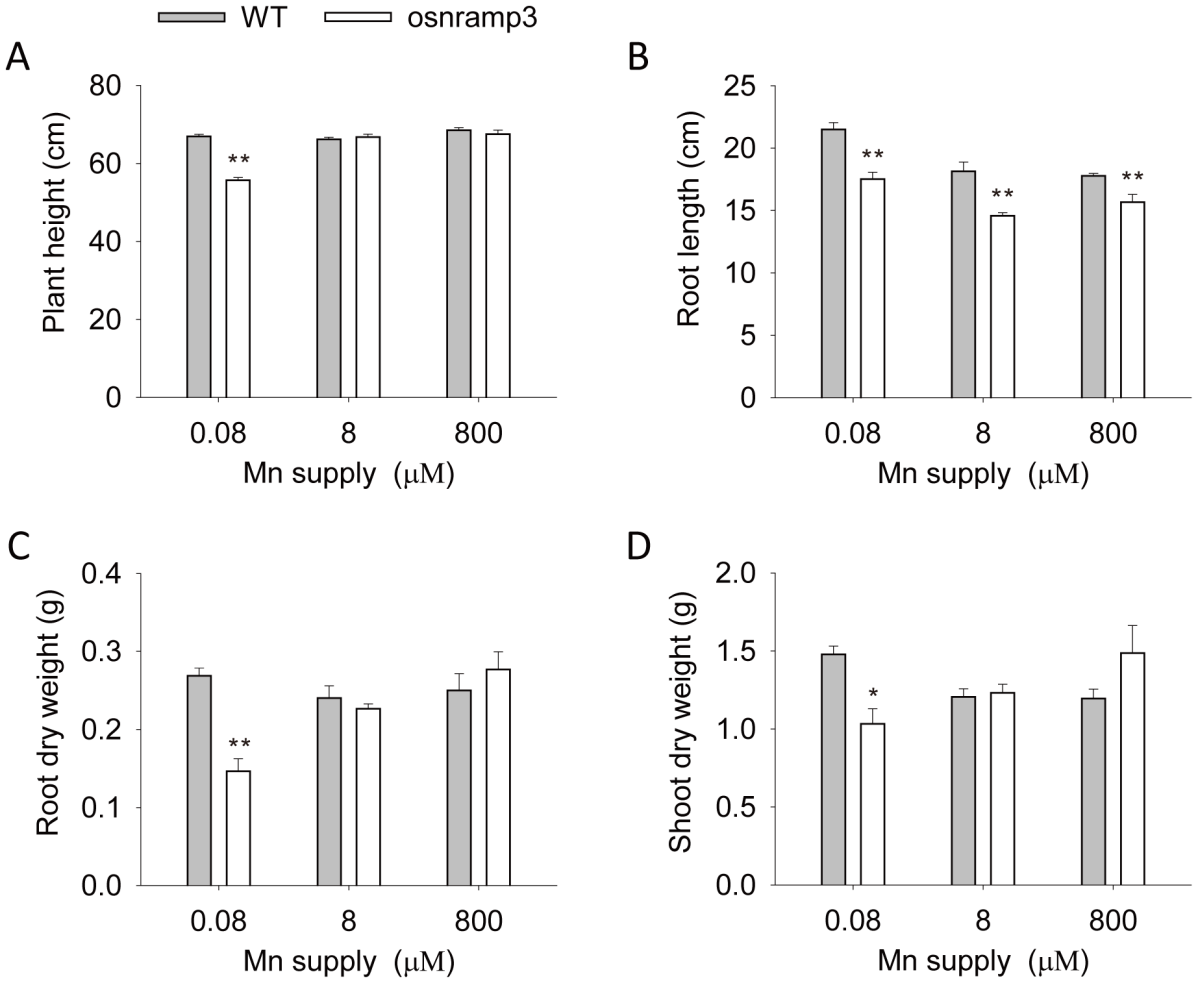
Effects of different Mn supplies on the growth of knockout plants of *OsNRAMP3*. The plants were cultivated hydroponically under normal conditions for two weeks and then shifted to three different Mn supplies for an additional two weeks. The plant heights (A) and root lengths (B) were investigated. Then the roots (C) and shoots (D) were harvested separately and their dry weights recorded. Data are means ± SD (n = 15). One and two asterisks indicate values that are significantly different from wild type at P<0.05 and P<0.01, respectively (by *t*-test).

After harvest, the plant height, root length and dry weight of samples were recorded. At low Mn (0.08 µM) condition, the plant heights and root lengths of *osnramp3* showed obvious decreases (Figure 3A and 3B); and dry weights of the roots and shoots in *osnramp3* were 54 and 70% of wild type, respectively (Figure 3C and 3D), which confirmed the observed phenotype (Figure 2). When supplied with medium (8 µM) or high (800 µM) Mn, root of *osnramp3* plants were shorter than that of wild type but there were no differences in plant height or dry weight.

### Metal Determination in Roots and Shoots

**Figure 4:**
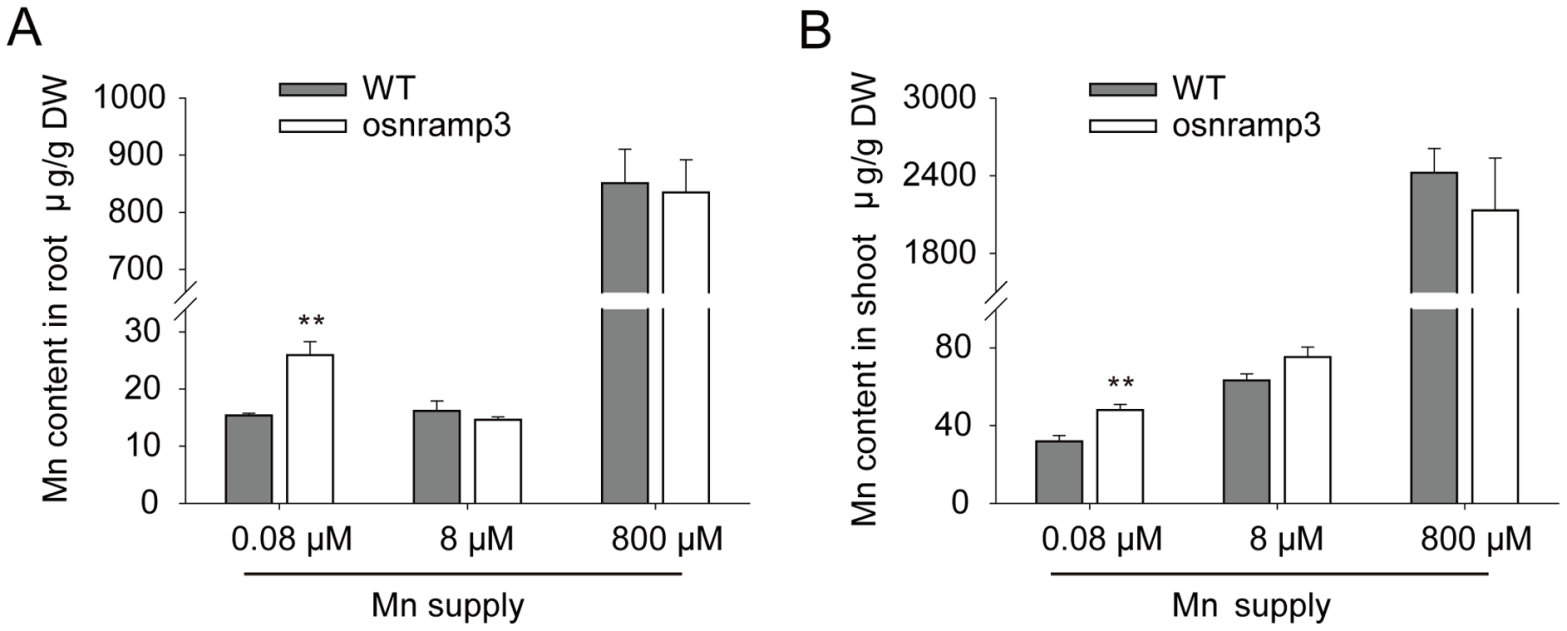
Determination of Mn concentration in wild type and *osnramp3* plants. Elemental analysis was performed by ICP-MS on roots (A) and shoots (B) of wild type or *osnramp3* plants grown for two weeks at three different Mn supplies after two weeks of normal conditional cultivation by hydroponics. Data are means ± SD (n = 3). One and two asterisks indicate values that are significantly different from wild type at P<0.05 and P<0.01, respectively (by *t*-test).

Since the knockout line of *OsNRAMP3* was sensitive to low Mn, we determined the Mn contents of roots and shoots of *osnramp3* and wild-type plants. At a 0.08 µM Mn supply, both roots and shoots of the knockout line accumulated significantly more Mn than the wild type (Figure 4A and 4B). There was no apparent difference in Mn contents between *osnramp3* and wild-type plants at higher Mn supplies. Absence of *OsNRAMP3* also did not affect magnesium (Mg), Ca, Fe, copper (Cu), zinc (Zn) and cadmium (Cd) contents of roots and shoots of rice plants cultivated under normal conditions (Figure S2).

### Knockout of *OsNRAMP3* Resulted in Disturbed Distribution of Mn in Leaves

**Figure 5:**
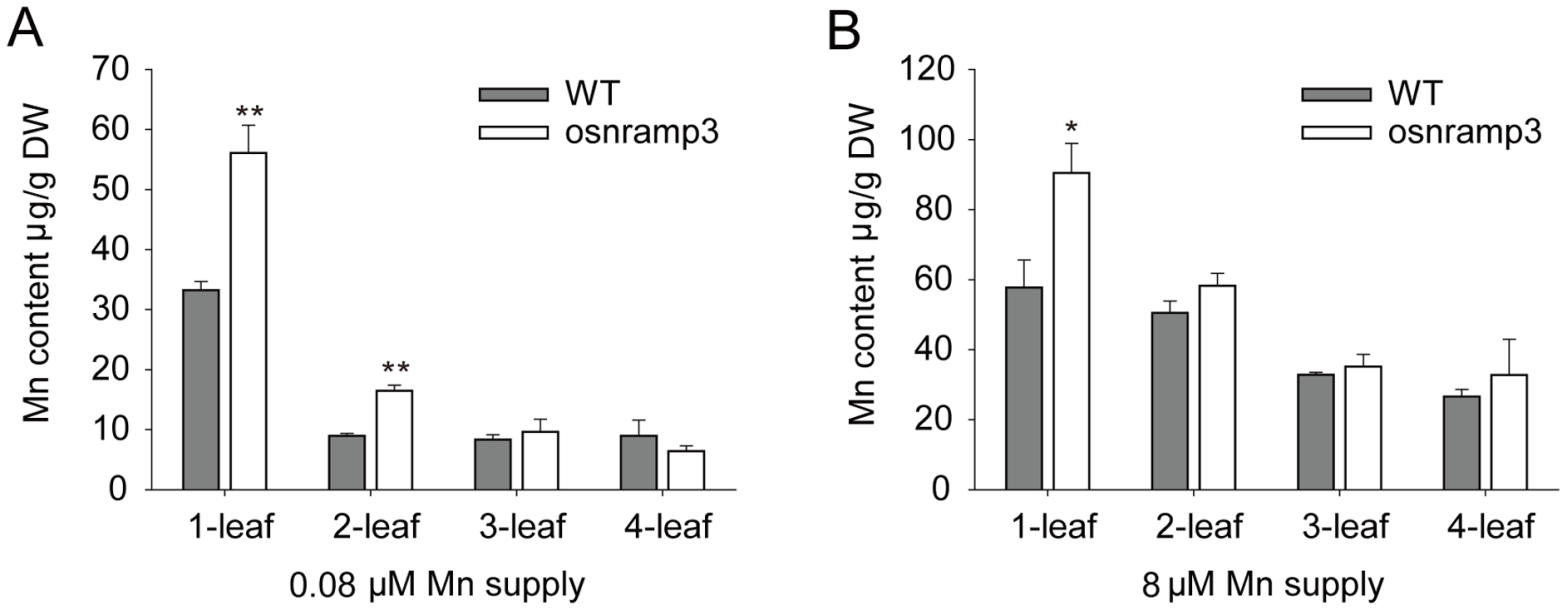
Metal analysis of different leaves in wild type and *osnramp3* plants. Different leaves were sampled from wild type and *osnramp3* plants grown for two weeks at 0.08 (A) or 8 (B) µM Mn supplies after two weeks of normal conditional cultivation by hydroponics and elemental analysis was performed by ICP-MS. The 1–4 leaves were harvested from the same tiller of wild type or *osnramp3* plants, and represented leaves from oldest to youngest respectively. Data are means ± SD (n = 3). One and two asterisks indicate values are significantly different from wild type at P<0.05 and P<0.01, respectively (by *t*-test).

It seemed contradictory that *osnramp3* mutant plants possessed higher Mn contents in roots and shoots but showed reduced growth under low Mn conditions compared with wild-type plants. To determine how this occurred, we next examined the distribution of Mn contents in different leaves for 0.08 or 8 µM Mn supplies. In general, Mn contents increased with leaf age in the treated and control plants. However, at a 0.08 µM Mn supply, *osnramp3* plants showed significantly higher Mn contents in the first and second leaves than wild-type plants; but there were no significant differences in third and fourth leaves (Figure 5A). Under 8 µM Mn conditions, higher Mn contents were also observed in first leaves of *osnramp3* compared with wild-type plants (Figure 5B); however, this difference was not observed in other leaves. The results here revealed that the absence of *OsNRAMP3* in rice caused the Mn accumulation in older leaves, suggesting that *OsNRAMP3* was important for Mn distribution in leaves.

In addition, the contents of Ca, Fe and Cd in older leaves were slightly, but not significantly, higher than that of wild type (Figure S3). There were no differences in Mg, Cu and Zn contents in corresponding leaves between wild type and *osnramp3* plants.

### Expression of *OsNRAMP3* was Specific to Vascular Bundles

**Figure 6:**
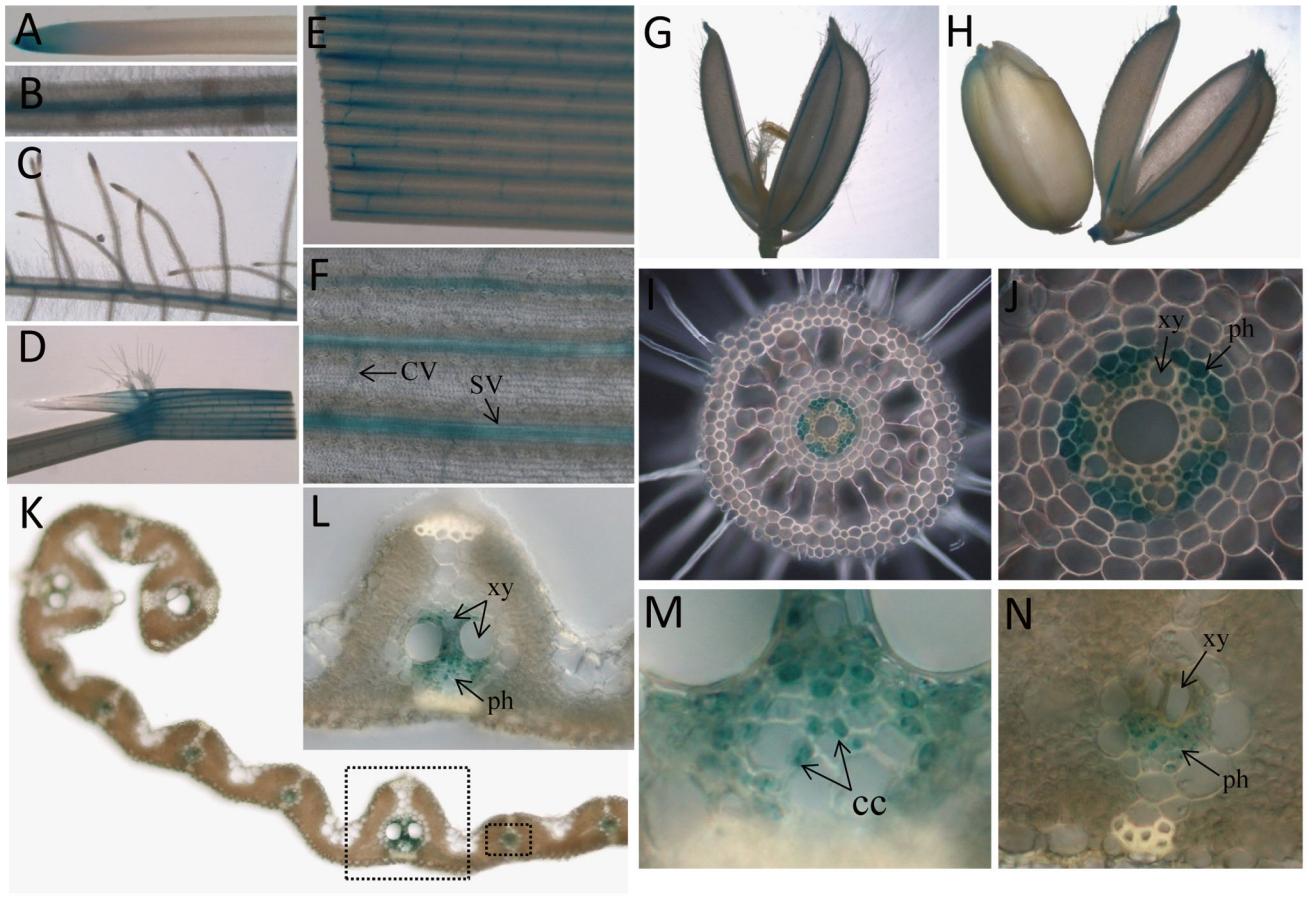
Histochemical staining of GUS activity in rice plants transformed with the construct *OsNRAMP3*-promoter:*GUS*. (A) Root tip; (B) mature root at 2 cm from tip; (C) lateral root; (D) leaf sheath, ligule and auricle; (E) leaf blade; (F) partly enlarged view of E; (G) hull at heading stage; (H) endosperm and hull at 25 d after heading; (I) transverse section of root, as described in B; (J) high-magnification of I; (K) transverse section of leaf blade, as described in E; (L) detail of a large vascular bundle from K; (M) enlarged view of phloem region of L; and (N) high-magnification of a small vascular bundle from K. All samples except hulls and endosperm were harvested from rice plants grown hydroponically for three weeks under normal conditions. CV: commissural vein; SV: small vascular bundle; LV: large vascular bundle; xy: xylem; ph: phloem; cc: companion cells.

To further investigate the expression pattern of *OsNRAMP3* in different tissues, a 2106-bp promoter region of *OsNRAMP3* was used to direct beta-glucuronidase (GUS) expression in rice plants. GUS activity was analyzed for different rice tissues. Although there was high GUS activity in various tissues, such as roots (Figure 6A, 6B and 6C), ligules and sheaths of leaves (Figure 6D), leaf blades (Figure 6E and 6F) and grain hulls (Figure 6G), it was specific to vascular bundles of these tissues. In addition, GUS activity was also found in root tips and dorsal vascular bundles of endosperm (Figure 6A and 6H). To determine the exact location of GUS activity, we made transverse sections of roots and leaves after GUS staining. High-magnification observation of roots showed that GUS activity was specific in phloem cells (Figure 6I and 6J). In leaves, GUS activity was mainly detected in phloem cells as also for roots (Figure 6K, 6L, 6M and 6N). An enlarged view of the phloem region revealed particularly strong GUS activity in companion cells (Figure 6M). In addition, some parenchyma cells between phloem and xylem also showed GUS activity in large vascular bundles (Figure 6L).

### *OsNRAMP3* Encodes a Plasma Membrane-localized Protein

**Figure 7:**
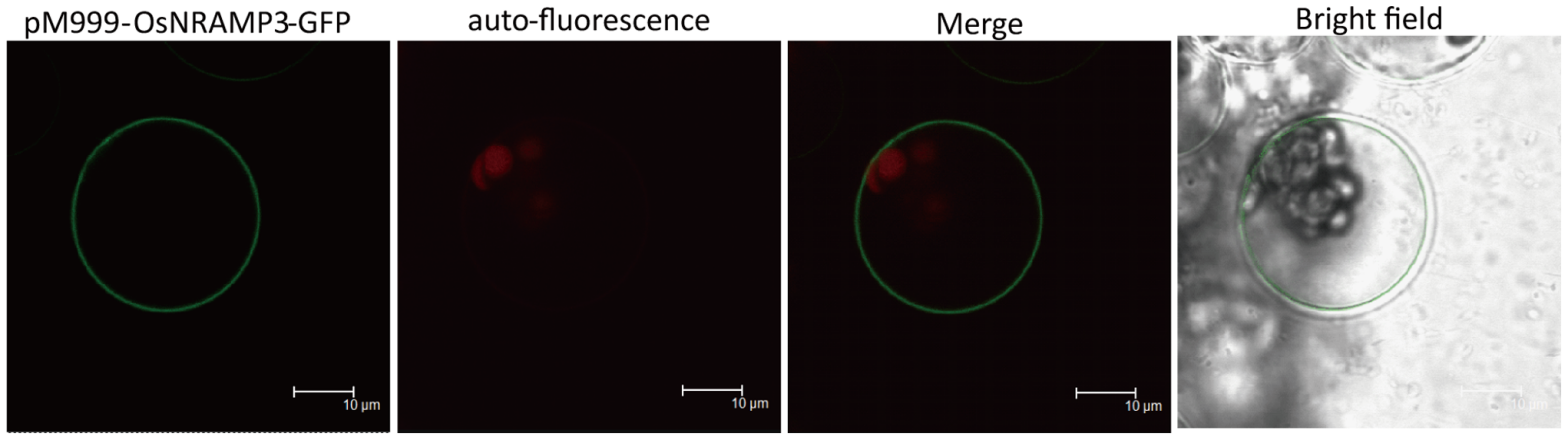
Subcellular localization of OsNRAMP3 protein. Subcellular localization of OsNRAMP3 protein was determined in *Arabidopsis* protoplasts. The confocal images were acquired using a confocal laser scanning microscope (TCS SP2; Leica).

To study the subcellular localization of the OsNRAMP3 protein, a vector expressing an *OsNRAMP3*–*GFP* fusion under the control of cauliflower mosaic virus (CaMV) 35S promoter was constructed and transformed into *Arabidopsis* protoplasts. The OsNRAMP3–GFP expression in the protoplasts was examined with confocal microscopy 16 h after transformation. The fluorescence showed that OsNRAMP3 was a plasma membrane-localized protein (Figure 7).

### OsNRAMP3 is a Functional Mn-influx Transporter

**Figure 8:**
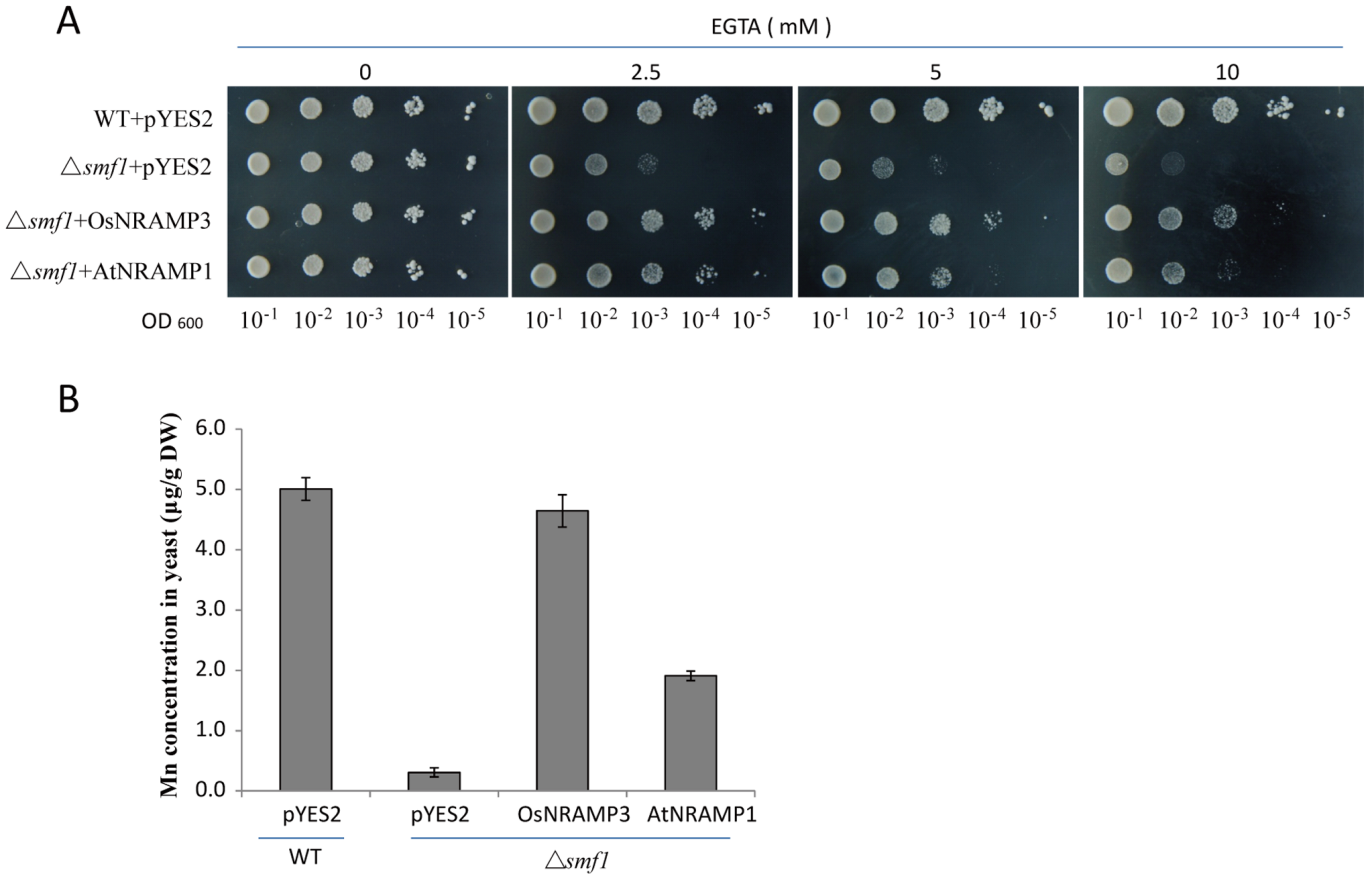
Complementation assay of *OsNRAMP3* on Mn-uptake deficient yeast mutant strain. (A) Yeast mutant *Δsmf1* and its wild type (WT) cells containing *pYES2* (vector), *OsNRAMP3* or *AtNRAMP1* (positive control) grown on synthetic defined (SD)-Ura plates with different EGTA supplies and 2% galactose. (B) Metal determination in *Δsmf1* and its wild-type cells containing *pYES2*, *OsNRAMP3* or *AtNRAMP1* grown in liquid SD-Ura culture with 0.2 µM Mn supplies and 2% galactose. Data are means ± SD (n = 4).

To determine the Mn transport activity of OsNRAMP3, we expressed *OsNRAMP3* in yeast mutant strain *Δsmf1*. Here *AtNRAMP1* was used as a positive control, as AtNRAMP1 has been shown to be a functional Mn transporter and can retrieve the growth of *Δsmf1* under Mn-limited conditions [5], [36]. The growth of yeast strains *Δsmf1* and its wild type transformed with *pYES2* (negative control), *OsNRAMP3* or *AtNRAMP1* were analyzed on Mn-limited medium which controlled by different concentration of ethylene glycol tetraacetic acid (EGTA). The results showed that *OsNRAMP3* significantly rescued the growth of *Δsmf1* when Mn was limited (Figure 8A), suggesting that OsNRAMP3 possessed high activity for transporting Mn. To further confirm this result, metal concentrations were analyzed in these strains. When *OsNRAMP3* was expressed in yeast strain *Δsmf1*, the Mn contents were almost retrieved to the level of wild type (Figure 8B), strongly indicating that OsNRAMP3 was a Mn-influx transporter.

## Discussion

### OsNRAMP3 is a Vascular Bundle-located Mn-influx Transporter Involved in Mn Translocation

NRAMP family genes were well known to be related to Mn transport and many of them which encoded plasma-membrane targeted proteins acted as high-affinity transporters to absorb Mn from outside: such as *SMF1* from yeast, *AtNRAMP1* from *Arabidopsis* and *OsNRAMP5* from rice [4]–[6]. In the present study, we identified *OsNRAMP3*, another NRAMP gene from rice, with the function of Mn transport. We demonstrated that knockout of *OsNRAMP3* resulted in serious reduction in growth of rice plants under low Mn conditions and this reduction could be rescued by higher Mn supply (Figure 2 and 3). Indeed, the contents and translocation of Mn in rice plants were obviously affected by the knockout of *OsNRAMP3* (Figure 4 and 5). Consistent with a role of *OsNRAMP3* in the Mn translocation are the following findings: (1) *OsNRAMP3* encoded a plasma membrane protein, based on the GFP fluorescence from OsNRAMP3–GFP fusion protein transformed into *Arabidopsis* protoplasts (Figure 7); (2) OsNRAMP3 was an influx Mn transporter, at least in yeast, based on the fact that *OsNRAMP3* can rescue the growth and increase Mn contents of yeast when expressed in yeast mutant *Δsmf1* (Figure 8); and (3) the expression of *OsNRAMP3* was specific to vascular bundles, especially in companion cells of phloem, based on the GUS staining analysis (Figure 6).

### Possible Mechanisms of OsNRAMP3 Involvement in Mn Translocation

In general, proteins encoded by NRAMP genes can be divided into two groups: plasma membrane-targeted and intracellular membrane-located proteins. The plasma membrane-targeted proteins from the NRAMP family contribute to uptake of Mn from medium as described above [5], [6]. The intracellular membrane-located proteins always act to retrieve Mn from vacuoles or other organelles: such as AtNRAMP3 and AtNRAMP4 from *Arabidopsis* [7], TcNRAMP3 and TcNRAMP4 from *Thlaspi caerulescens* [8], and LeNRAMP1 and LeNRAMP3 from tomato [9]. In this study, OsNRAMP3 showed difference: *OsNRAMP3* encoded a plasma membrane protein (Figure 7) but was not involved in Mn uptake. The knockout mutant of *OsNRAMP3* was highly sensitive to Mn deficiency (Figure 2C), which were similar to the characteristics of *OsNRAMP5* described by Sasaki et al. [6]. However, *OsNRAMP3* expression was restricted to vascular bundles rather than the exodermis and endodermis of roots (Figure 6), indicating a different function from *OsNRAMP5*. In addition, knockout of *OsNRAMP3* resulted in Mn contents increasing in roots and shoots of *osnramp3* mutant plants (Figure 4), suggesting that *OsNRAMP3* was involved in Mn translocation but not Mn uptake.

Young leaves of *osnramp3* mutant plants appeared to be withered compared with wild-type plants under low Mn conditions; and the withered appearance could be retrieved by higher Mn supply (Figure 2C), suggesting that the withered leaves of *osnramp3* were deficient in the Mn required to maintain growth. However, *osnramp3* plants accumulated more Mn in both roots and shoots compared with wild-type plants (Figure 4), which seemed contradictory. Most probably as feedback of signals of lacking Mn in withered leaves, the Mn uptake system of *osnramp3* plants was enhanced to absorb more Mn from outside, which caused increased Mn contents in roots and shoots of *osnramp3* compared with wild type. Consistent with that, further studies showed that the Mn content of each leaf of *osnramp3* plants was not lower than corresponding leaves of wild-type plants (Figure 5A). This suggested that the symptoms of lacking Mn in withered leaves were caused by disturbed distribution of Mn rather than general Mn shortage. GUS analysis in leaves showed that *OsNRAMP3* was specifically expressed in phloem cells and some parenchyma cells between phloem and xylem (Figure 6M and 6N), implying that the disturbed distribution of Mn was probably due to sequestrating Mn into vascular bundles of *osnramp3* plants. As OsNRAMP3 is an influx Mn transporter, and parenchyma cells in vascular bundles always operate the loading or unloading of ion for xylem [37], [38], the Mn transported via xylem was probably unable to leave the xylem due to the absence of OsNRAMP3 in knockout lines. However, this suggestion requires more supporting evidence.

Phloem always plays important roles in remobilization of various ions in shoots, such as Zn, Fe, Cu and Mn [39]–[41]. In barley, it was reported that the transpiration stream has little effect on Mn translocation to the youngest leaf, whereas the strong effect on Mn translocation in older leaves [42], implying that major Mn concentration in young leaf is probably transported through the phloem. In the present study, *OsNRAMP3* encoded a Mn-influx transporter and was mainly expressed in phloem cells, suggesting that *OsNRAMP3* may contribute to remobilization of Mn in shoots. The increased Mn contents in *osnramp3* plants mainly occurred in older not younger leaves under low Mn conditions (Figure 5A). Coincidentally, in rice plants, the expression of *OsNRAMP3* in leaves slightly increased with leaf aging (Figure 1B). This implied that *OsNRAMP3* played an important role in leaf Mn distribution. Together with the phenotype that only young leaves of *osnramp3* plants were sensitive to Mn deficiency, the results here strongly suggested that *OsNRAMP3* operated the Mn remobilization from old to young leaves via phloem cells.

In rice, *OsYSL2* was reported to be a phloem-located metal transporter responsible for long-distance transport of Fe and Mn, especially in seeds [16], [41]. Similarly to *OsYSL2*, the expression of *OsNRAMP3* was also detectable in vascular bundles of endosperm and hulls, suggesting that *OsNRAMP3* was required in these tissues. Confirming whether *OsNRAMP3* is involved in transporting Mn into seeds requires more detailed study. In roots, the expression of *OsNRAMP3* was specific to phloem cells and root tip cells (Figure 6A and 6J). Coincidently, the root tip of knockout plants of *OsNRAMP3* became necrotic under low Mn conditions (Fig. 2C, panel e). We investigated the Mn contents in different root regions at different distances from the root tip in rice cv. Zhonghua 11 (data not shown). The root tip possessed the highest Mn contents of the different root regions, suggesting that Mn played an important role in growth of root tips. However, because of serious necrosis in root tips of *osnramp3* plants under low Mn conditions, it is difficult to determine the Mn contents in *osnramp3* root tips. To determine whether *OsNRAMP3* is related to root tip Mn contents and how *OsNRAMP3* affects growth of root tips will require further study.

In conclusion, OsNRAMP3 is a vascular bundle-localized influx Mn transporter that is involved in Mn translocation in rice plants. Knockout of *OsNRAMP3* disturbed the transport of Mn into young leaves from old leaves and vascular bundle cells, resulting in high sensitivity to low Mn conditions. It is worth noting that when we finished our experiments, we noticed a related but independent work by Yamaji et al. [43], in which *OsNRAMP3* was also described as a Mn transporter contributing to Mn distribution in shoots.

## Supporting Information

Figure S1 **Response of** ***OsNRAMP3*** **expression to different Mn concentrations.** The plants were cultivated hydroponically under normal conditions for two weeks and then shifted to different Mn supplies for an additional two weeks, and then harvested for RNA extraction.
(TIF)

Figure S2 **Concentration of various metals in roots and shoots of wild type and** ***osnramp3*** **plants.** The plants were cultivated hydroponically under normal conditions for four weeks. Roots (A) and shoots (B) were harvested separately. Before sampling, roots were washed twice with deionized water. All samples were dried at 80°C for 3 d.
(TIF)

Figure S3 **The distribution of various metals in different leaves.** Mg (A), Ca (B), Fe (C), Cd (D), Cu (E) and Zn (F) contents were determined in different leaves of wild type and *osnramp3* plants cultivated under normal conditions for four weeks. The 1–4 leaves were harvested from the same tiller of wild type or *osnramp3* plants, and represented the oldest to youngest leaves, respectively.
(TIF)
