# Supplementary figures and images for: OsNRAMP3 Is a Vascular Bundles-Specific Manganese Transporter That Is Responsible for Manganese Distribution in Rice
Source: PLoS One. 2013 Dec 31;8(12):e83990. doi: 10.1371/journal.pone.0083990 (PMC3877151; doi:10.1371/journal.pone.0083990)

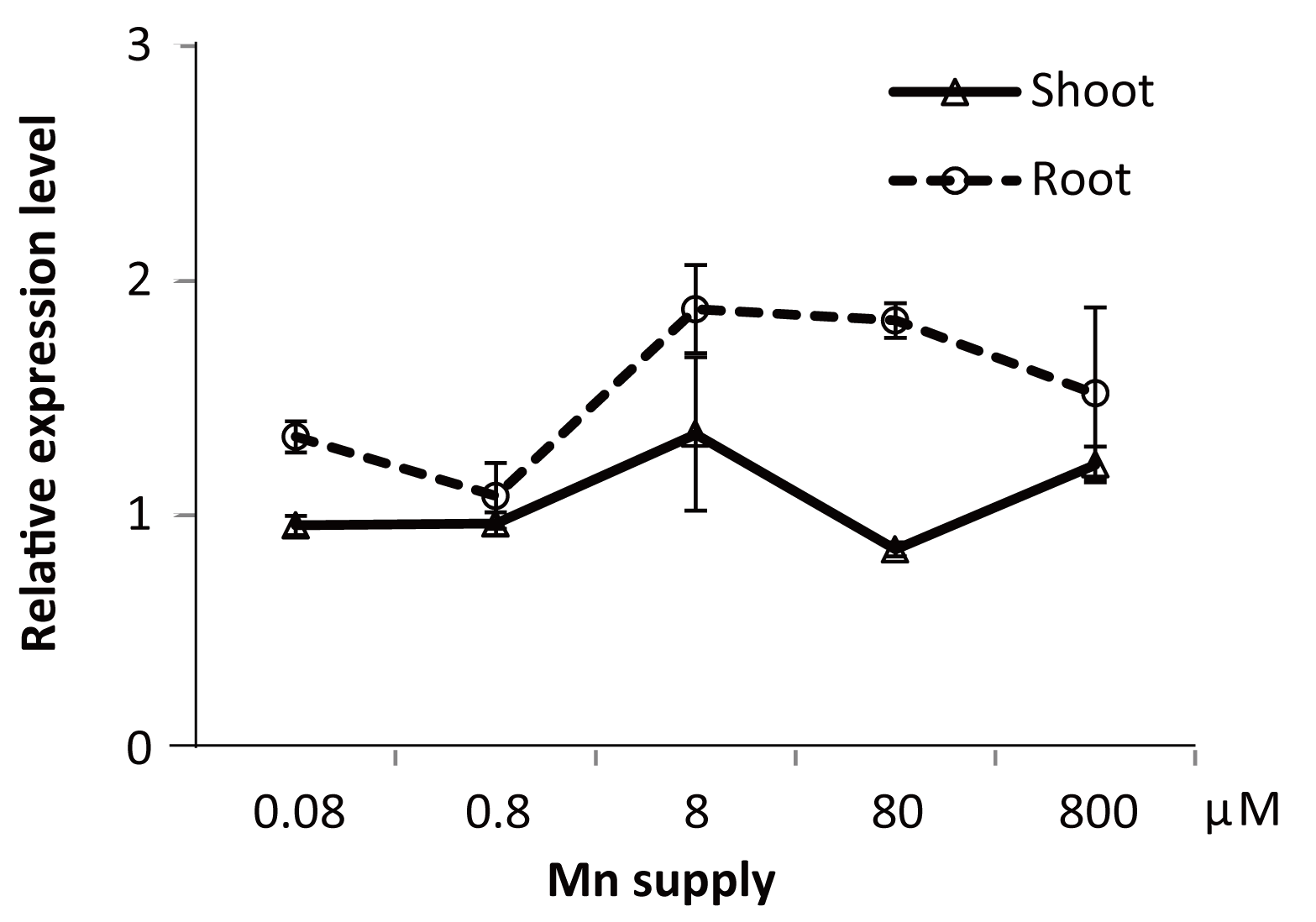

Supplement: Figure S1 — Response of OsNRAMP3 expression to different Mn concentrations. The plants were cultivated hydroponically under normal conditions for two weeks and then shifted to different Mn supplies for an additional two weeks, and then harvested for RNA extraction. (TIF) [file pone.0083990.s001.tif]

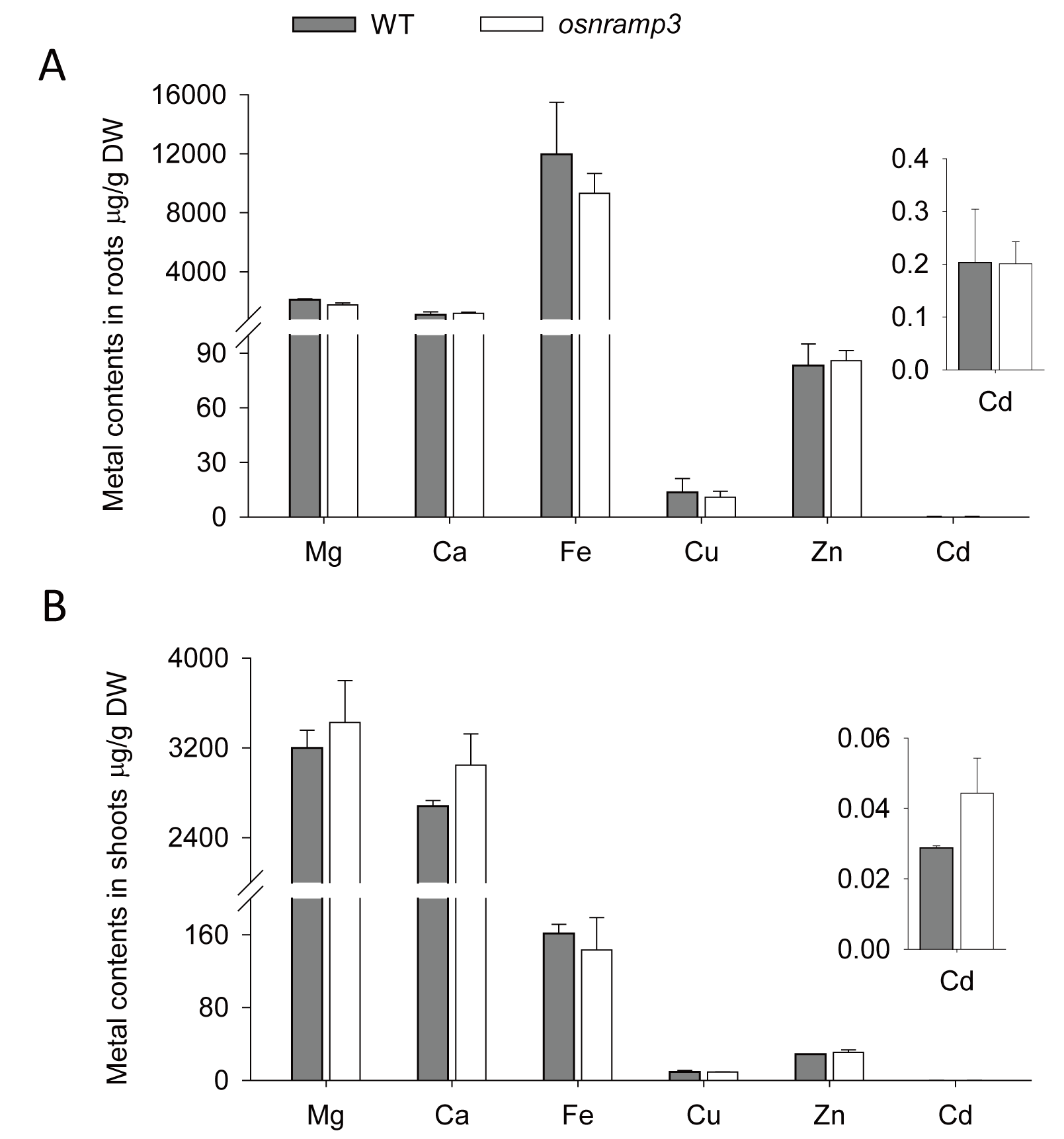

Supplement: Figure S2 — Concentration of various metals in roots and shoots of wild type and osnramp3 plants. The plants were cultivated hydroponically under normal conditions for four weeks. Roots (A) and shoots (B) were harvested separately. Before sampling, roots were washed twice with deionized water. All samples were dried at 80°C for 3 d. (TIF) [file pone.0083990.s002.tif]

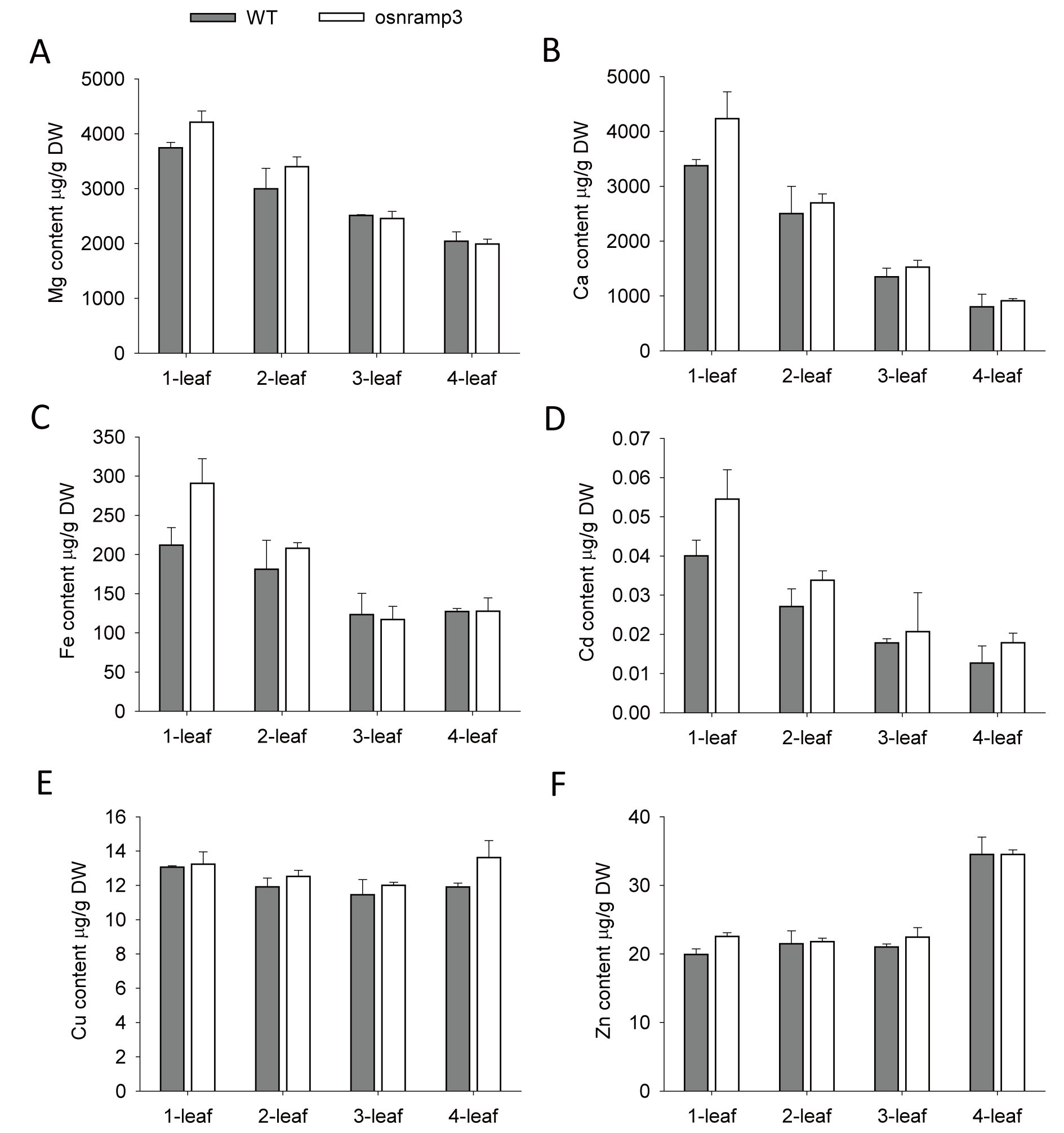

Supplement: Figure S3 — The distribution of various metals in different leaves. Mg (A), Ca (B), Fe (C), Cd (D), Cu (E) and Zn (F) contents were determined in different leaves of wild type and osnramp3 plants cultivated under normal conditions for four weeks. The 1–4 leaves were harvested from the same tiller of wild type or osnramp3 plants, and represented the oldest to youngest leaves, respectively. (TIF) [file pone.0083990.s003.tif]
